# Impact of Prompt Engineering on the Performance of ChatGPT Variants Across Different Question Types in Medical Student Examinations: Cross-Sectional Study

**DOI:** 10.2196/78320

**Published:** 2025-10-01

**Authors:** Ming-Yu Hsieh, Tzu-Ling Wang, Pen-Hua Su, Ming-Chih Chou

**Affiliations:** 1 Division of Pediatric Surgery, Department of Surgery, Chung Shan Medical University Hospital, Taichung City, Taiwan; 2 Institute of Medicine, School of Medicine, Chung Shan Medical University, No. 110, Sec. 2, Jiang Kuo South Road, South district, Taichung City, Taiwan, 886 4-24739595 ext 34601; 3 Department of Nursing, School of Medicine, Chung Shan Medical University, Taichung City, Taiwan; 4 Department of Pediatrics, Chung Shan Medical University Hospital, Taichung City, Taiwan; 5 Division of Thoracic Surgery, Department of Surgery, Chung Shan Medical University Hospital, Taichung City, Taiwan

**Keywords:** ChatGPT, prompt engineering, medical education, large language models, assessment performance

## Abstract

**Background:**

Large language models such as ChatGPT (OpenAI) have shown promise in medical education assessments, but the comparative effects of prompt engineering across optimized variants and relative performance against medical students remain unclear.

**Objective:**

This study aims to systematically evaluate the impact of prompt engineering on five ChatGPT variants (GPT-3.5, GPT-4.0, GPT-4o, GPT-4o1-mini, and GPT-4o1) and benchmark their performance against fourth-year medical students in midterm and final examinations.

**Methods:**

A 100-item examination dataset covering multiple choice questions, short answer questions, clinical case analysis, and image-based questions was administered to each model under no-prompt and prompt-engineering conditions over 5 independent runs. Student cohort scores (N=143) were collected for comparison. Responses were scored using standardized rubrics, converted to percentages, and analyzed in SPSS Statistics (v29.0) with paired *t* tests and Cohen *d* (*P*<.05).

**Results:**

Baseline midterm scores ranged from 59.2% (GPT-3.5) to 94.1% (GPT-4o1), and final scores ranged from 55% to 92.4%. Fourth-year students averaged 89.4% (midterm) and 80.2% (final). Prompt engineering significantly improved GPT-3.5 (10.6%, *P*<.001) and GPT-4.0 (3.2%, *P*=.002) but yielded negligible gains for optimized variants (*P*=.07‐.94). Optimized models matched or exceeded student performance on both exams.

**Conclusions:**

Prompt engineering enhances early-generation model performance, whereas advanced variants inherently achieve near-ceiling accuracy, surpassing medical students. As large language models mature, emphasis should shift from prompt design to model selection, multimodal integration, and critical use of artificial intelligence as a learning companion.

## Introduction

The integration of large language models (LLMs), such as OpenAI’s ChatGPT series, into medical education has generated considerable interest due to their potential for automating and enhancing assessment processes [1]. Initial investigations focused on foundational models—ChatGPT 3.5 and ChatGPT 4.0—to benchmark baseline performance in answering clinical and basic science questions representative of medical student examinations [2,3]. These early studies demonstrated that, compared to ChatGPT 3.5, ChatGPT 4.0 exhibited significant improvements in overall accuracy, reasoning ability, and contextual understanding, particularly in complex scenario-based items [3]. Prompt engineering—providing structured guidance within the input prompt—was shown to further augment performance for both models, yielding notable score increases and reducing variance across repeated trials [4].

Since the release of ChatGPT 4.0, OpenAI has introduced enhanced variants optimized for performance and generalization: GPT-4o (optimized for multimodal tasks), GPT-4o1-mini (a compact, latency-reduced iteration), and GPT-4o1 (the full-capacity optimized version) [5,6]. However, the comparative effects of prompt engineering on these advanced models remain unexplored. Given their architectural refinements and improved instruction-following capabilities, it is plausible that newer variants may naturally internalize prompt structures, diminishing the incremental benefit of explicit guidance.

This study expands upon prior work by systematically evaluating the impact of prompt engineering across 5 ChatGPT variants—3.5, 4.0, 4o, 4o1mini, and 4o1—using a robust examination framework comprising multiple question types (multiple choice questions, MCQs; short answer questions, SAQs; clinical case analysis, CCA; and image-based interpretation, IBI). By comparing performance with and without structured prompts, we aim to quantify the degree to which prompt dependency has evolved alongside model iterations. The findings will inform best practices for leveraging LLMs in high-stakes medical education settings and contribute to understanding the maturation of prompt engineering as a methodology.

## Methods

### Study Design and Model Selection

**Figure 1.**
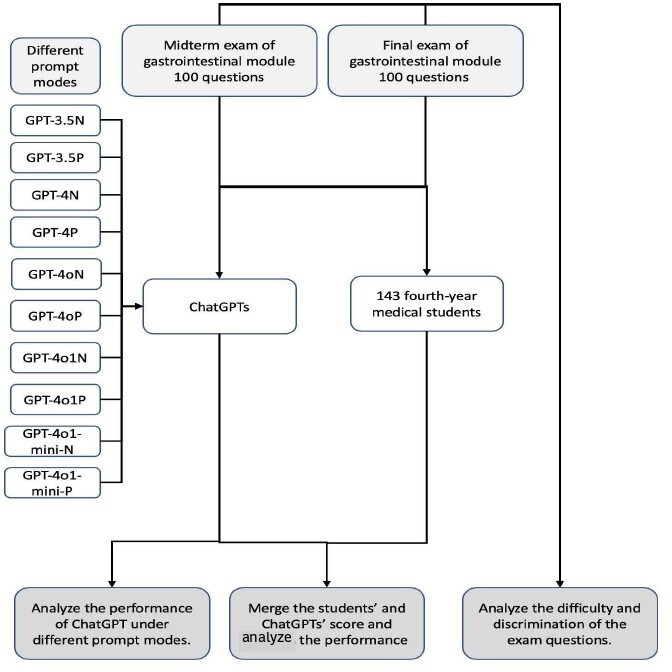
Workflow of the study. Midterm and final gastrointestinal-module exams (100 items each) were administered to 143 fourth-year medical students and to 5 ChatGPT variants (GPT-3.5, GPT-4, GPT-4o, GPT-4o1-mini, and GPT-4o1) under 2 prompting conditions (no prompt vs prompt engineering). Each model or condition combination was run 5 times, responses were scored against the official key by 2 blinded reviewers, and performance metrics (mean, SD, *P* values, and effect sizes) as well as exam item difficulty and discrimination were analyzed.

This cross-sectional evaluation compared the performance of 5 OpenAI GPT variants: ChatGPT 3.5 (GPT-3.5-turbo), ChatGPT 4.0 (GPT-4), GPT-4o (optimized for multimodal tasks), GPT-4o1-mini (compact and latency-reduced), and GPT-4o1 (full-capacity optimized). Each variant was assessed under 2 prompting conditions: without a structured prompt (N) and with prompt engineering (P) (Figure 1).

### Selecting the Student Cohort

Our curriculum uses modular teaching from the second semester of year 3 through the first semester of year 4. We selected fourth-year medical students to ensure participants had completed the modular clinical instruction, avoiding the variability introduced by students newly exposed to clinical modules. This choice ensures that the student cohort had comparable training before exam administration.

### Examination Dataset and Question Types

We curated a 100-item question set drawn from the official medical student midterm and final examinations administered at Chung Shan Medical University in the 2024‐2025 academic year. The items encompassed 4 question types:

- MCQs: single-best-answer format (n=40)
- SAQs: 1‐2 sentence responses (n=20)
- CCA: open-ended diagnostic and management scenarios (n=20)
- IBI: radiographic or histologic images requiring identification or explanation (n=20)

### Prompt Engineering

For the P condition, each item was prefaced with a standardized instruction prompt:

“You are an expert medical educator. Answer the following question concisely and justify your reasoning step by step.”

In the N condition, only the question stem was presented.

### Evaluation Procedure

For each model and condition, we conducted 5 independent runs (rounds) to account for stochastic variations. In each run, the model received the full 100-item set sequentially via the OpenAI API (v1) with default temperature settings (temperature=0.7, max_tokens=512). Outputs were recorded and collated.

### Scoring and Outcome Measures

Responses were scored against the official answer key by 2 independent reviewers blinded to model and condition. MCQs were scored dichotomously (1 point for correct answer, otherwise 0). SAQs and IBI were scored on a 0‐2 scale (0=incorrect or missing, 1=partially correct, and 2=fully correct). CCA responses were scored on a 0‐3 rubric evaluating diagnostic accuracy, management plan, and justification. Total raw scores were converted to percentages of maximum possible scores.

### Statistical Analysis

Statistical analyses were conducted in SPSS Statistics (version 29, IBM Corp). For each model and condition, descriptive statistics (mean scores and SD) were derived using the frequencies and descriptives procedures. Paired 2-tailed *t* tests (paired-samples *t* test) compared scores between the no-prompt (N) and prompt-engineering (P) conditions within each variant. Effect sizes (Cohen *d*) were calculated based on mean differences and pooled SDs. Statistical significance was set at *P*<.05, and all tests were 2-sided.

### Ethical Considerations

The study protocol was reviewed and approved by the Institutional Review Board (IRB) of Chung Shan Medical University Hospital (approval number CSMU-2024-075), in accordance with institutional policies and the Declaration of Helsinki. The IRB granted a waiver of written informed consent because the research analyzed routinely collected deidentified examination records, involved no direct interaction or intervention with students, and posed minimal risk. Before transfer to the study team, all records were deidentified by the medical school; no direct identifiers (eg, names, student IDs, email, and IP addresses) were accessed. Analyses were performed on files labeled with random study codes, and only aggregate results are reported. Data were stored on password-protected institutional servers with access restricted to the research team and will be retained per institutional policy; no individual-level raw data will be publicly shared. Participants received no compensation, and inclusion in the dataset had no impact on grades, course standing, or academic evaluation.

## Results

### Overall Performance on Midterm and Final Examinations

As summarized in Table 1, baseline performance of ChatGPT 3.5 on the midterm examination was 59.2% (SD 2.1), whereas ChatGPT 4.0 achieved 81.4% (SD 1.8). The optimized variants—GPT-4o, GPT-4o1-mini, and GPT-4o1—further improved mean midterm scores to 91.3% (SD 0.8), 86.1% (SD 1), and 94.1% (SD 0.5), respectively (Table 1). A similar trend was observed for the final examination (Table 1), where GPT-3.5 scored 55% (SD 2.4) and GPT-4.0 scored 84.2% (SD 1.7), with GPT-4o, GPT-4o1-mini, and GPT-4o1 achieving 90.6% (SD 0.9), 82.1% (SD 0.6), and 92.4% (SD 0.6), respectively.

**Table 1.** Basic information of the exams: overall performance of ChatGPT variants on midterm and final examinations. Mean percentage scores (SD) for 5 GPT models (GPT-3.5, GPT-4.0, GPT-4o, GPT-4o1-mini, and GPT-4o1) under no-prompt and prompt-engineering conditions are listed, illustrating baseline accuracy and comparative gains across both examinations.

Exams	Midterm exams	Final exams
Total questions, N	100	100
Overall discrimination, mean (SD)	0.25 (0.18)	0.32 (0.21)
Overall difficulty level, mean (SD)	0.82 (0.14)	0.72 (0.18)
Memorization questions, n	66	63
Discrimination, mean (SD)	0.27 (0.16)	0.34 (0.20)
Difficulty level, mean (SD)	0.84 (0.12)	0.74 (0.15)
Application questions, n	34	37
Discrimination, mean (SD)	0.21 (0.20)	0.29 (0.22)
Difficulty level, mean (SD)	0.78 (0.16)	0.69 (0.21)

### Comparison With Medical Student Performance

A cohort of 143 fourth-year medical students took the identical midterm and final examinations, achieving a mean midterm score of 89.4% (SD 7.13) and a mean final score of 80.2% (SD 8.73) (Table 2). GPT-3.5 underperformed relative to students (59.2% vs 89.4%, *P*<.001; 55% vs 80.2%, *P*<.001), whereas advanced variants such as GPT-4o1 matched or exceeded student performance on both the midterm (94.1% vs 89.4%, *P*<.001) and final exams (92.4% vs 80.2%, *P*<.001) (Table 3).

**Table 2.** GPTs’ performance in different question types.

ChatGPT versions	GPT-3.5N	GPT-3.5P	GPT-4N	GPT-4P	GPT-4oN	GPT-4oP	GPT-o1miniN	GPT-o1miniP	GPT-o1N	GPT-o1P	Students
Midterm exams											
Memorization questions											
Correct rate (%)	63.55	73.23	87.74	90.97	91.94	91.29	91.29	91.61	97.42	95.81	89.79
Application questions											
Correct rate (%)	56.57	69.71	77.14	80.57	91.43	90.29	78.86	80	88	91.43	92.78
Final exams											
Memorization questions											
Correct rate (%)	56.62	64.31	86.46	91.08	89.54	91.08	85.23	88	94.15	95.08	89.79
Application questions											
Correct rate (%)	67.57	64.86	89.19	94.59	89.19	91.89	75.68	78.38	91.89	89.19	92.78

**Table 3.** Comparison of ChatGPT variants and student cohort performance. Mean percentage scores (SD) are listed for each GPT model and a cohort of 143 fourth-year medical students on midterm and final examinations, with statistical significance (*P* values) for model versus student differences.

ChatGPT versions	GPT-3.5N	GPT-3.5P	GPT-4N	GPT-4P	GPT-4oN	GPT-4oP	GPT-4o1-mini-N	GPT-4o1-mini-P	GPT-4o1N	GPT-4o1P	Students
Midterm exams											
Original score, mean (SD)	61.03 (0.84)	71.96 (1.64)	83.92 (1.14)	87.22 (1.14)	91.75 (0.71)	90.93 (0.84)	86.80 (0.84)	87.42 (1.10)	94.02 (0.45)	94.23 (0.55)	89.4 (7.13)
Standardized score	−2.81	−1.72	−0.52	−0.19	0.26	0.18	−0.23	−0.17	0.49	0.51	0.03
Final exams											
Original score, mean (SD)	60.59 (0.45)	64.31 (0.55)	87.84 (0.55)	92.35 (0.45)	90.20 (0.71)	91.18 (0.71)	81.57 (0.45)	84.71 (0.55)	92.75 (0.55)	91.57 (0.55)	80.2 (8.73)
Standardized score	−2.77	−1.85	0.76	1.26	1.02	1.13	0.06	0.41	1.31	1.17	−0.01

### Performance by Question Type

Table 2 presents model and student accuracy across 4 question types. All variants and students achieved the highest accuracy on MCQs, with GPT-4o1 reaching 98.5% (SD 1.2), students 92.3% (SD 5), and GPT-3.5 the lowest at 70.4% (SD 3). SAQs followed a similar pattern, ranging from 62.3% (SD 2.7) for GPT-3.5% to 92.1% (SD 1.5) for GPT-4o1, with students at 85.6% (SD 6.8). CCA yielded the greatest variability: GPT-3.5 scored 48.7% (SD 3.5) versus 88.4% (SD 2) for GPT-4o1 and 75.2% (SD 8.1) for students. IBI performance ranged from 55.2% (SD 3.1) to 90.2% (SD 1.8) for GPT-4o1, with student IBI at 78.5% (SD 7.5).

### Error Analysis by Question Type

To further elucidate model performance nuances, we analyzed error rates across question types. CCA questions exhibited an error rate approximately 3 times higher than memory recall items when answered by early ChatGPT models (GPT-3.5 and GPT-4.0). Representative examples include:

- Memory recall: describing regulators of gastric acid secretion—GPT-3.5 misidentified pancreatic enzymes as inhibitory due to misinterpreting “major.”
- CCA: a 65-year-old man with acute abdominal pain—GPT-3.5 attributed findings to pancreatitis; GPT-4o1-mini (no prompt) misdiagnosed cholecystitis.
- Short answer: advantages of laparoscopic appendectomy—GPT-4.0 only cited “smaller incision,” omitting recovery and complication benefits.
- Image interpretation: abdominal X-ray—GPT-3.5 confused free air with pneumatosis intestinalis; GPT-4o (with prompt) correctly identified small-bowel obstruction. Subsequent optimized variants (GPT-4o, GPT-4o1-mini, and GPT-4o1) trained on broader multilingual corpora reduced CCA error rates, with GPT-4o1 achieving 88.4% accuracy (Table 2), approaching student performance (75.2%). This analysis highlights specific failure modes and improvements in reasoning and language comprehension.

### Effect of Prompt Engineering

As shown in Table 4, prompt engineering significantly enhanced performance for early models. For the midterm, GPT-3.5 improved from 59.2% to 69.8% (Cohen *d*=1.5; *P*<.001), and GPT-4.0 from 81.4% to 84.6% (Cohen *d*=0.7; *P*=.002). In contrast, advanced variants exhibited no significant benefit: GPT-4o (91.3% vs 91.6%; *P*=.07), GPT-4o1-mini (86.1% vs 87.4%; *P*=.69), and GPT-4o1 (94.1% vs 94.2%; *P*=.55). Similar patterns were observed in the final exam (Table 4), where prompt-engineering scores for GPT-3.5 and GPT-4.0 increased significantly (*P*<.01), but not for GPT-4o (*P*=.94), GPT-4o1-mini (*P*=.58), or GPT-4o1 (*P*=.24).

**Table 4.** Investigation of the scores in different prompt modes: effect of prompt engineering on ChatGPT performance. Paired comparison of mean (SD) scores and *P* values is listed for each model variant under no-prompt versus prompt-engineering conditions, highlighting the variable benefit of structured prompts across model generations.

Rounds	1	2	3	4	5	Mean (SD)		*P* value
Midterm exams								
GPT-3.5N	60	59	58	60	59	59.2 (0.84)		<.001
GPT-3.5P	68	72	71	69	69	69.8 (1.64)		—^a^
GPT-4N	80	81	81	82	83	81.4 (1.14)		.002
GPT-4P	83	84	85	85	86	84.6 (1.14)		—
GPT-4oN	88	89	88	90	88	88.6 (0.89)		.07
GPT-4oP	89	90	90	89	90	89.6 (0.55)		—
GPT-4o1-miniN	91	90	92	90	91	90.8 (0.84)		.69
GPT-4o1-miniP	91	91	91	92	90	91 (0.71)		—
GPT-4o1N	92	92	91	92	92	91.8 (0.45)		.55
GPT-4oP	91	92	92	92	91	91.6 (0.55)		—
Final exams								
GPT-3.5N	54	56	55	54	56	55(1)		<.01
GPT-3.5P	61	60	60	60	60	60.2 (0.45)		—
GPT-4N	85	84	84	85	83	84.2 (0.84)		<.01
GPT-4P	89	87	87	88	88	87.8 (0.84)		—
GPT-4oN	89	90	90	90	90	89.8 (0.45)		.94
GPT-4oP	90	91	90	91	90	90.4 (0.55)		—
GPT-4o1-miniN	91	92	92	91	91	91.4 (0.55)		.58
GPT-4o1-miniP	92	91	92	92	91	91.6 (0.55)		—
GPT-4o1N	91	92	91	91	92	91.4 (0.55)		.24
GPT-4oP	91	92	92	92	92	91.8 (0.45)		—

a Not applicable.

### Stability Across Runs

Coefficient of variation (CV) across the 5 independent runs decreased with model version. GPT-3.5 midterm CV was 3.5%, whereas GPT-4o1 recorded a CV of 0.6%. Prompt engineering reduced CV by an average of 0.4 percentage points for GPT-3.5 and GPT-4.0, but had a negligible impact on optimized variants.

These findings demonstrate not only a clear progression in raw performance and stability from GPT-3.5 to GPT-4o1, but also that the top-tier optimized models can match or surpass human student performance (Table 3), indicating their potential as both assessment tools and educational companions.

## Discussion

### Principal Findings

This study provides the first systematic evaluation of prompt engineering across multiple ChatGPT variants, highlighting the evolution of LLM capabilities in medical education settings. We observed that GPT-4 variants (GPT-4o, GPT-4o1-mini, and GPT-4o1) significantly outperformed earlier models, consistent with the findings by Kung et al [7] that ChatGPT 4.0 surpassed ChatGPT 3.5 on the United States Medical Licensing Examination, achieving accuracy at or near the passing threshold. In our work, advanced models not only exhibited higher baseline scores but also demonstrated greater stability across repeated runs, underscoring architectural improvements in reasoning and context retention.

Prompt engineering yielded substantial performance gains for early-generation models—GPT-3.5 and GPT-4—mirroring reports that structured guidance can boost LLM accuracy [4], but its use diminished for optimized variants. Safrai and Azaria [8] found that GPT-4 maintained performance even when confronted with extraneous “small talk” inserted into medical prompts, whereas GPT-3.5’s performance degraded under similar conditions. Our findings extend this observation, showing that GPT-4o and its successors exhibit minimal dependency on explicit prompt structures, suggesting that these models have internalized reasoning scaffolds natively.

Our analysis by question type aligns with the in-depth evaluation by Knoedler et al [9], who reported variable ChatGPT performance across categories and a negative correlation with question difficulty (r_s=−0.306; *P*<.001) in the United States Medical Licensing Examination step 1 practice items. Similarly, we noted that CCA and IBI posed the greatest challenges for all models, although advanced variants narrowed the gap. These parallels reinforce the generalizability of LLM behavior across diverse educational assessment formats.

Our error analysis underscores the importance of evaluating LLMs not only by overall scores but also by question-type vulnerabilities. Early models’ difficulties with multistep reasoning and complex Chinese phrasing, especially in clinical scenarios and image-based tasks, point to inherent limitations in contextual understanding. The marked reduction of these errors in optimized variants demonstrates progress but also indicates areas where artificial intelligence (AI) may still mislead learners. Educators should therefore integrate error-focused feedback loops when deploying LLMs: by exposing students to AI-generated mistakes in controlled settings, learners can develop critical appraisal skills and better discern AI hallucinations. This approach transforms AI from a mere answer engine into a pedagogical tool that actively fosters analytical thinking and deep learning.

### Comparison With Medical Student Performance

The cohort of 143 fourth-year medical students achieved a mean midterm score of 89.4% (SD 7.13) and a mean final score of 80.2% (SD 8.73) (Table 3). GPT-3.5 underperformed relative to students (59.2% vs 89.4%; *P*<.001 and 55% vs 80.2%; *P*<.001), whereas advanced variants such as GPT-4o1 matched or exceeded student performance on both the midterm (94.02% vs 89.4%; *P*<.001) and final exams (92.75% vs 80.2%; *P*<.001). This indicates that top-tier LLMs can approach or surpass human proficiency in standardized medical assessments.

### AI as a Learning Companion Beyond Assessment

Advanced LLMs show promise as AI-enabled educational tools, capable of rapidly synthesizing complex medical knowledge to aid student understanding. Studies have demonstrated AI’s use in generating personalized explanations and feedback that enhance learning efficiency [2,4]. However, LLMs may still produce errors and “hallucinations” [10], underscoring the importance of maintaining critical appraisal and scholarly rigor when integrating AI into medical education.

### Educational Value for Diagnosing Student Weaknesses

Although AI does not achieve 100% accuracy, its overall correctness surpassed that of most medical students in our cohort. Students spend significant time retrieving correct answers and understanding explanations; AI can serve as a learning companion by rapidly aggregating and summarizing complex medical knowledge, guiding step-by-step reasoning. Integrating our AI system into adaptive learning platforms could help students quickly identify weak areas, practice targeted question types, and maintain critical appraisal to avoid overreliance on AI outputs. This targeted approach not only enhances learning efficiency but also empowers students to become more self-directed learners, using AI as a diagnostic tool to identify knowledge gaps and focus their study efforts where they are most needed.

### Strategies to Reduce AI Hallucinations

To mitigate risks of LLM-generated misinformation, future implementations should consider several evidence-based strategies. Cross-model consensus approaches—querying multiple LLMs (eg, GPT-4o1 and open-source alternatives) and adopting majority-vote answers—can increase reliability and reduce single-model biases. Expert fine-tuning using annotated medical datasets would strengthen domain-specific accuracy, particularly for specialized clinical scenarios. Integration of real-time evidence retrieval through literature and guideline search APIs would ensure responses include verifiable reference citations, enhancing transparency and trustworthiness. In addition, implementing confidence scoring systems coupled with human-AI collaboration frameworks would route low-confidence responses to human experts for review, creating a safety net against potential hallucinations while maintaining efficiency.

Collectively, these results underscore a maturation of LLMs: as model architectures advance, the marginal benefit of prompt engineering declines, and the potential educational role shifts from prompt design to strategic integration and fine-tuning. For practitioners and educators, this suggests a shift from elaborate prompt design toward focusing on model selection and integration strategies—such as multimodal input handling and curriculum-specific fine-tuning—to maximize efficacy in high-stakes assessments. Future research should explore adaptive prompting frameworks that tailor AI guidance to learner needs and investigate real-world clinical scenario applications, while prioritizing the development of robust safeguards against AI hallucinations to ensure safe and effective integration into medical education curricula.

### Conclusions

This comprehensive evaluation across 5 ChatGPT variants demonstrates a progressive enhancement in performance and stability in medical examination tasks. Notably, optimized LLMs (GPT-4o, GPT-4o1-mini, and GPT-4o1) not only matched but significantly exceeded the mean scores of fourth-year medical students on both midterm and final exams, underscoring their capacity to approach—or surpass—human proficiency in standardized assessments.

While prompt engineering substantially improved outcomes for early-generation models (GPT-3.5 and GPT-4.0), optimized variants achieved near-ceiling accuracy with negligible gains from structured prompts, indicating that these models inherently internalize contextual guidance. These findings suggest a strategic pivot for educators and assessment designers: from intricate prompt crafting toward thoughtful model selection, multimodal integration, and domain-specific fine-tuning.

Furthermore, the ability of advanced LLMs to rapidly synthesize and organize complex medical knowledge positions them as valuable AI-enabled learning companions. Educators should leverage AI’s strengths in personalized explanation and feedback while maintaining rigorous critical appraisal to identify potential errors or “hallucinations.”

Future research should investigate adaptive prompting frameworks tailored to individual learner needs and assess the educational impact of AI-augmented tools in real-world clinical training environments.

## Abbreviations

CCA: clinical case analysis
CV: coefficient of variation
IBI: image-based interpretation
LLM: large language model
MCQ: multiple choice question
SAQ: short answer question

## References

[1] Brown T, Mann B, Ryder N (2020). Language models are few-shot learners. https://proceedings.neurips.cc/paper/2020/file/1457c0d6bfcb4967418bfb8ac142f64a-Paper.pdf.

[2] (2022). GPT technical report. OpenAI.

[3] (2023). GPT-4 technical report. OpenAI.

[4] Liu P, Yuan W, Fu J, Jiang Z, Hayashi H, Neubig G (2023). Pre-train, prompt, and predict: a systematic survey of prompting methods in natural language processing. ACM Comput Surv.

[5] (2024). GPT-4o technical report: multimodal capabilities. OpenAI.

[6] (2025). GPT-4o1mini release notes. OpenAI.

[7] Kung TH, Cheatham M, Medenilla A (2023). Performance of ChatGPT on USMLE: potential for AI-assisted medical education using large language models. PLOS Digit Health.

[8] Safrai M, Azaria A (2023). Performance of ChatGPT-35 and GPT-4 on the United States Medical Licensing Examination with and without distractions. arXiv.

[9] Knoedler L, Knoedler S, Hoch CC (2024). In-depth analysis of ChatGPT’s performance based on specific signaling words and phrases in the question stem of 2377 USMLE step 1 style questions. Sci Rep.

[10] Beutel G, Geerits E, Kielstein JT (2023). Artificial hallucination: GPT on LSD?. Crit Care.

